# A genetically encoded bioluminescent indicator for illuminating proinflammatory cytokines

**DOI:** 10.1016/j.mex.2016.06.001

**Published:** 2016-07-07

**Authors:** Sung Bae Kim, Takeaki Ozawa, Yoshio Umezawa

**Affiliations:** a Research Institute for Environmental Management Technology, National Institute of Advanced Industrial Science and Technology (AIST), 16-1 Onogawa, Tsukuba 305-8569, Japan; b Department of Chemistry, School of Science, The University of Tokyo, 7-3-1 Hongo, Bunkyo-Ku, Tokyo 113-0033, Japan

**Keywords:** Genetically encoded bioluminescent indicator based on protein splicing, Bioluminescence, Protein splicing assay (PSA), Luciferase, Cytokine, nuclear factor-κB (NF-κB), Tumor necrosis factor-α (TNF-α), Nuclear trafficking

## Abstract

We introduce a method to evaluate the activities of cytokines based on the nuclear transport of NF-κB. A pair of bioluminescent indicators was made for conferring cytokine sensitivity to cervical carcinoma-derived HeLa cells. The principle is based on reconstitution of split fragments of *Renilla reniformis* luciferase (RLuc) by protein splicing with a DnaE intein from *Synechocystis* sp. PCC6803. The bioluminescence intensity of thus reconstituted RLuc in the HeLa cells was used as a measure of the activities for cytokines. With the present method, we evaluated the activities of various cytokines based on the nuclear transport of NF-κB in human cervical carcinoma-derived HeLa cells carrying the indicators. The present approach to evaluating the activities of cytokines may provide a potential clinical value in monitoring drug activity and directing treatment for various diseases related with NF-κB. The method highlights the experimental procedure from our original publications, *Anal. Biochem.* 2006, 359, 147–149 and *Proc. Natl. Acad. Sci. U. S. A.* 2004, 101, 11542.

The summary of the method is:

• Cytokine activities are determined within 2 h after stimulation.
• Temporarily inactivated split-luciferase fragments are reconstituted by protein splicing.
• Nucleartrafficking of NF-κB was illuminated for gauging the ligand-driven activity.

## Graphical abstract

**Figure:**
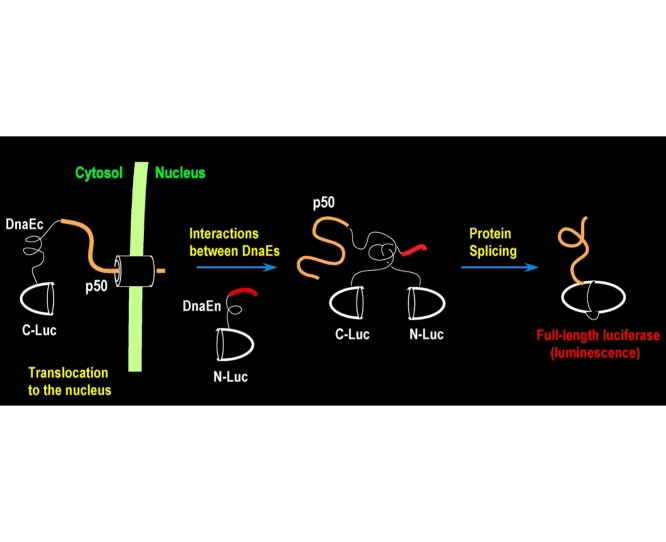

## Method details

### Introduction

**Fig. 1:**
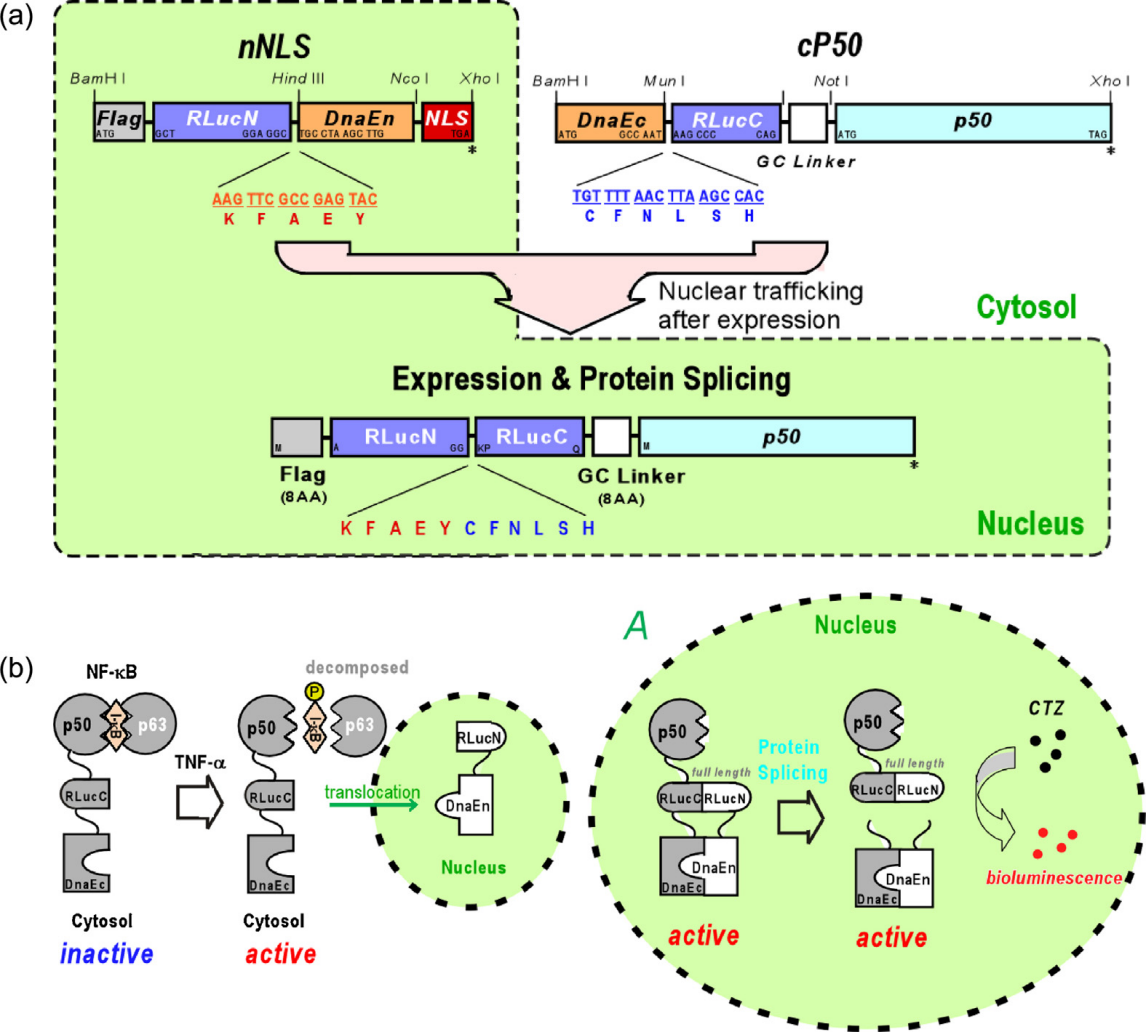
(A) Schematic structure of cDNA constructs of the indicators. The constructs encoding nNLS and cP50 are subcloned into a mammalian expression vector, and cotransfected into a mammalian cell. After expression, nNLS and cP50 are sequestered in the nucleus and the cytosol, respectively. A cytokine triggers nuclear trafficking of cP50 and the consequent protein splicing in the nucleus. GC linker: a cDNA coding flexible amino acids, GGGGSGGG. Flag epitope: a cDNA coding DYKDDDDK. Nuclear localization signal (NLS): a cDNA coding three consecutive repeat of DPKKKRKV. Additional sequences at the boundaries between RLuc and DnaE are shown on the bars. (B) Strategy for the detection of the nuclear trafficking of NF-κB. When NF-κB is stimulated by a pro-inflammatory cytokine, it releases the phosphorylated IκBs and is translocated into the cellular nucleus and binds with a specific DNA binding site to express specific proteins such as anti-inflammatory proteins. Inset *A* highlights the protein splicing reaction in the nucleus. In the nucleus, interaction between the N- and C-terminal fragments of split-*Renilla* luciferase (RLuc) causes protein splicing with a naturally split DnaE intein (a catalytic subunit of DNA polymerase III; 15 kD). A full length RLuc is reconstituted by the self-catalytic protein splicing. Subsequently, the bioluminescence intensities from the reconstituted RLuc are measured with a luminometer. IκB, p50, and p65 refer to inhibitor-κB, p50 and p65 subunits of NF-κB, respectively. The N- and C-terminal fragments of split-RLuc were abbreviated as ‘N’ and ‘C’.

The pro-inflammatory cytokines bind with their specific receptors on the plasma membrane of cells and finally activate nuclear factor-kappa B (NF-κB), which is deeply related to various human diseases including cancers, aging, diabetes, neurodegenerative disorders, and inflammatory diseases [1], [2], [3], [4], [5]. In this method, we describe a method to quantitatively determine inflammatory activities of cytokines within 3 h, based on the cytokine-induced nuclear transport of nuclear factor-kappa B (NF-κB) as illustrated in Fig. 1. Upon cytokine stimulation, the subunit of NF-κB, p50, is translocated into the nucleus, where the N- and C-terminal fragments of split-RLuc are spontaneously reconstituted with the help of a naturally split DnaE intein (a catalytic subunit of DNA polymerase III from the genome of *Synechocystis* sp. PCC6803 (*Ssp* genome); 15 kD), called a protein-fragment splicing assay (PSA). The optical intensity reflects the activities of cytokines. This imaging strategy highlights the experimental procedure of our precedent publications [6], [7].

### Materials

• Cytokines: Human tumor necrosis factor-α (TNF-α); Human oncostatin M (OSM); Human interleukin-1β (IL-1β); Human leukemia inhibitory factor (LIF) (Sigma); IL-1 receptor antagonist (IL-1RA).
• Reagents for genetic engineering: a lipofection reagent (TransIT-LT1, Mirus); a mammalian expression vector (pcDNA 3.1(+)); cDNA encoding p50 of NF-κB; cDNA encoding RLuc.
• Immunocytochemistry-related reagents: mouse anti-Flag antibody (Sigma); mouse anti-NF-κB P50 antibody (Abcam); Cy-5-conjugated secondary antibody (Jackson); paraformaldehyde (PFA) (Sigma); fish skin gelatin (FSG) (Sigma); Mowiol solution (Aldrich); Sytox Green (Molecular Probes).
• Buffers: a TBST buffer (Tris–HCl buffer with saline and Triton X-100); a phosphate buffered saline (PBS) buffer.
• Cell culture and assay reagents: human uterine cervical carcinoma-derived HeLa cells; Dulbecco’s modified eagle’s medium (DMEM; Sigma); cholesterol-free fetal bovine serum (FBS; Gibco); penicillin-streptmycin (P/S); *Renilla* luciferase assay kit (Promega), a Bradford reagent (Pierce).

### Design of the cDNA constructs

**Fig. 2:**
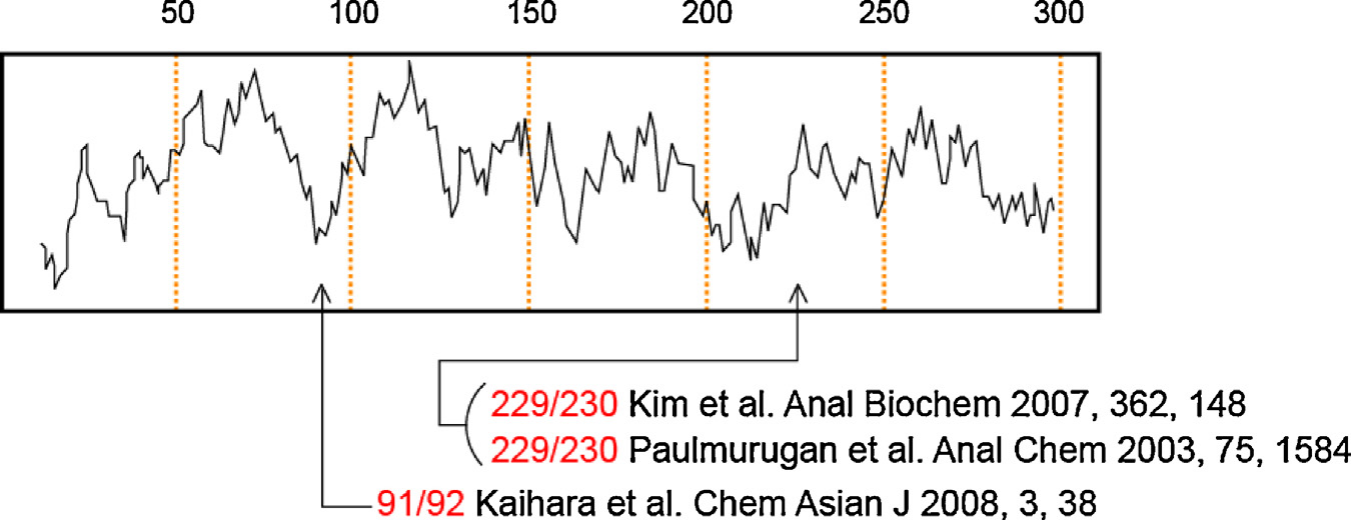
A hydrophilicity search of RLuc revealing a hydrophilic region in the middle of the sequence. The sequence was projected by the scale of Kyte and Doolittle [8]. The arrows show known dissection sites for bioassays in literatures [10], [11], [12].

The unique bioluminescent indicator is designed as follows (Fig. 1, Fig. 2).

1. Download the amino acid sequence of RLuc (Protein Databank assess number: 2PSD) from a public database, National Center for Biotechnology Information (NCBI).
2. Determine the hydrophilicity scale through pasting the full amino acid sequences of RLuc into the web window of ExPASy Proteomics service (Swiss Institute of Bioinfomatics; SIB) for obtaining the scale of Kyte and Doolittle [8].^1^
3. Choose a potential dissection site in the remarkably hydrophilic region in the middle of the scale (Fig. 2).^2^
4. Split the amino acid sequence of RLuc at the identified dissection site (e.g., between 229 and 230 AA), and link the corresponding N- and C-terminal fragments of RLuc to fabricate the N- and C-terminal fragment set of split-DnaE, respectively (Fig. 1).
5. Further fuse the sequences with a pair of proteins of interest, i.e., a nuclear localization signal (3 × NLS, three consecutive repeat of DPKKKRKV) and a subunit of NF-κB called p50, to the C-terminal ends of the above fragment set, respectively.
6. Add the amino acid sequences (“KFAEY” and “CFNLSH”) for an efficient protein splicing between the fusions to the splicing junctions of the N- and C-terminal fragments of DnaE, respectively.^3^ The N- and C-terminal fragment set may be called nNLS and cP50, respectively. The expressed N- and C-terminal components may be called nNLS and cP50, respectively.
7. One may link a “Flag” tag (DYKDDDDK) as an epitope to the N-terminal end of nNLS. A flexible GS linker (GGGGSGGG) may be inserted to the domain interfaces excepting the splicing junctions for flexibility of the fusions.

### Fabrication of cDNA constructs

Based on the molecular design above, one may conduct polymerase chain reactions (PCRs) to fabricate the corresponding cDNA constructs using the adequate primer sets as follows.

1. Design a series of primer sets on the basis of the molecular design of Fig. 1 to add unique enzyme sites to each segment: e.g., the 5′- and 3′-terminal ends of cDNA encoding p50 may be modified to carry *Not*I and *Xho*I sites with the primer set of AAAGCGGCCGCATGGCAGAAGATGATCCATAT (forward primer), and AAACTCGAGTTACATGGTTCCATGCTTCAT (reverse primer).
2. Run a series of PCRs with the designed primer set and a template to generate the cDNA segments encoding the following domains: i.e., the C-terminal domains of split-RLuc (RLuc-C, 230-311 AA) and DnaE (DnaE-C); the N-terminal domains of split-RLuc (RLuc-N, 1-229 AA) and DnaE (DnaE-N); P50; and NLS, as shown in Fig. 1A.
3. Ligate and subclone the cDNA constructs into a mammalian expression vector pcDNA 3.1(+).The plasmids may be named pnNLS and pcP50.
4. Confirm fidelity of the cDNA sequences in the vectors with a DNA sequencer, e.g., BigDye Terminator Cycle Sequencing kit and a genetic analyzer ABI Prism310 (PE Biosystems).

### An immunocytochemistry study for confirming the nuclear trafficking of cP50

**Fig. 3:**
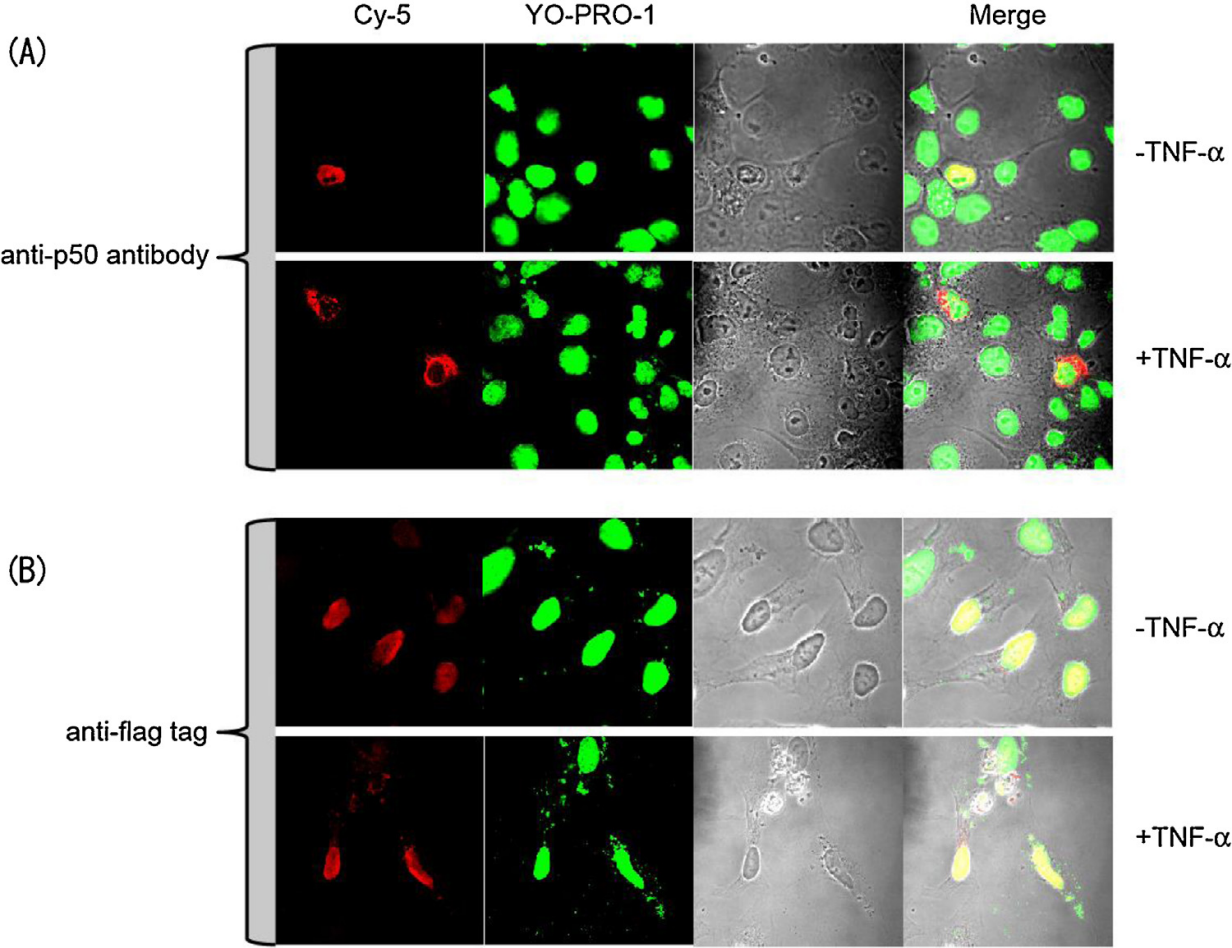
Immunocytochemical evaluation of the localization of the fusion proteins. The localizations of the nNLS and cP50 were recognized by anti-Flag or anti-NF-κB P50 antibodies, respectively, and stained with Cy-5-conjugated secondary antibodies (left column). The nuclei were stained with Sytox Green (middle column). The right column shows the merged images. In the first and second rows, the locations of the cP50 were imaged, whereas in the third and fourth rows, the localizations of nNLS were imaged. “TNF-α (+)” and “TNF-α (−)” represent the optical image after stimulation or mock-stimulation of TNF-α, respectively.

The indicator components, nNLS and cP50, are located in the nucleus and the cytosol, respectively. Cytokine-activated cP50 is translocated into the nucleus, where it meets nNLS and exerts a self-catalytic protein splicing. This working mechanism is confirmed with the following immunocytochemistry study (Fig. 3).^4^

1. Culture HeLa cells in the medium with Dulbecco’s modified eagle’s medium (DMEM; Sigma) supplemented with 10% cholesterol-free fetal bovine serum and 1% penicillin-streptmycin (P/S).
2. Subculture HeLa cells on thin cover glasses (0.7 × 10^5^ cells/slide) and transfected with pnNLS or pcP50.
3. Further incubate the cells for 12 h^5^ and stimulate the cells with a cytokine (50 ng/mL TNF-α^6^) or vehicle (phosphate buffered saline (PBS) alone) for 3 h.^7^
4. Fix the cells on the cover glasses with a 3% paraformaldehyde (PFA) solution.
5. Block the cells on the cover glasses with 0.2% fish skin gelatin (FSG) and then incubate with a mouse anti-NF-κB P50 antibody (Abcam) or a mouse anti-Flag antibody (Sigma).
6. Wash the primary antibody with a TBST buffer (Tris–HCl buffer with saline and Triton X-100) for 5 min. Repeat it three times.
7. Incubate the cells on the cover glasses with Cy-5-conjugated secondary antibody (Jackson) for 30 min and wash them two times with the TBST buffer.
8. Further stain the nuclei of the cells with Sytox Green (Molecular Probes) and wash them 3 times with the TBST buffer.
9. Fix the cover glasses on a larger micro slide glass (76 × 26 mm) using a Mowiol solution (Aldrich).
10. Record the fluorescence images using a confocal laser-scanning microscope (LSM510, Zeiss) fitted with a band-pass filter (514–535 nm) for Sytox Green and a long-pass filter (665 nm) for Cy-5.

### A cell-based in vitro study for illuminating the activities of proinflamatory cytokines

**Fig. 4:**
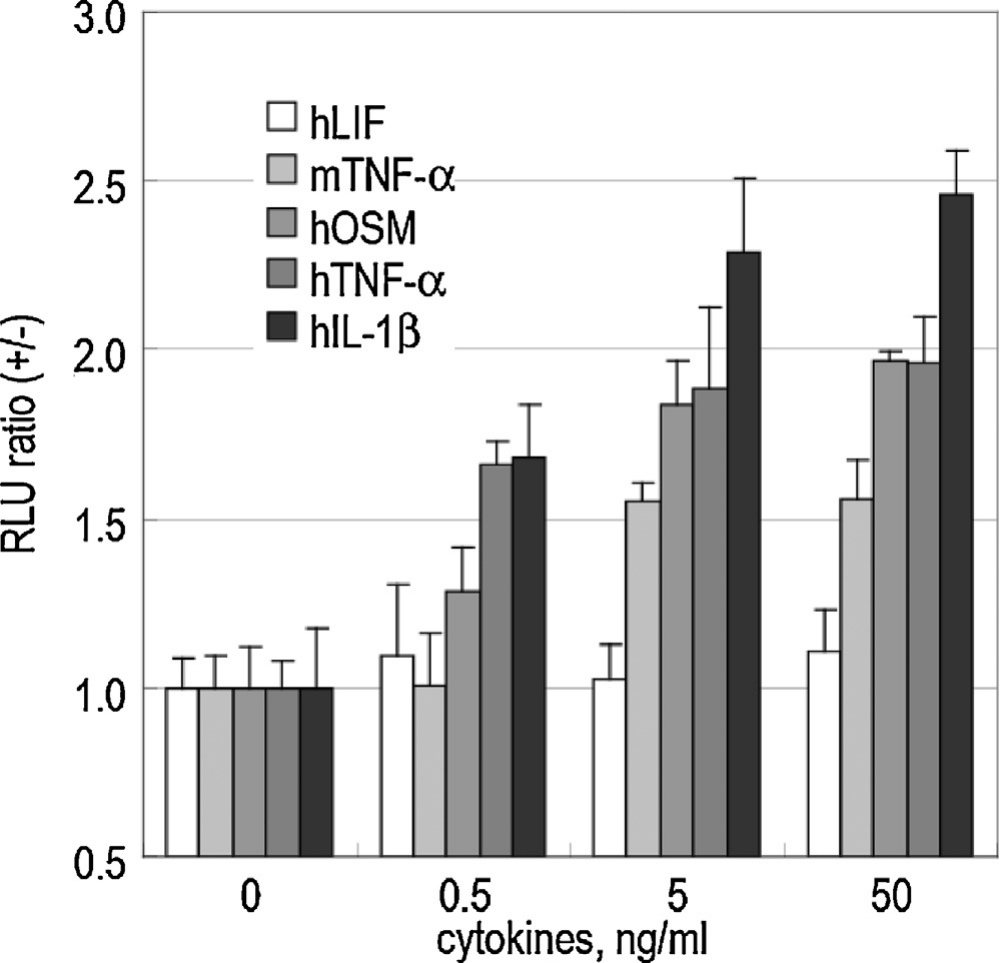
Cytokine concentration dependence of the luminescence intensity from the HeLa cells carrying pcDRc-p50 and pcRDn-NLS. The cotransfected cells were stimulated with each concentration of hLIF, murine TNF-α (mTNF-α), hOSM, human TNF-α (hTNF-α), and hIL-1β. The luminescence intensities were determined 2 h after the stimulation (*n* = 3).

Activities of proinflamatory cytokines are illuminated as follows (Fig. 4):

1. Culture HeLa cells in DMEM supplemented with 10% fetal bovine serum (FBS) and 1% P/S at 37 °C in 5% CO_2_.
2. Replace the medium with DMEM supplemented with 10% cholesterol-free fetal bovine serum and 1% P/S.
3. Transfect HeLa cells cultured in 12-well plates with pnNLS and pcP50 using a transfection reagent, TransIT-LT1 (Mirus).
4. Incubate in DMEM supplemented with 10% steroid-free FBS and 1% P/S for 12 h.
5. Stimulate the cells in each well with differing concentrations of cytokines (0.5–50 ng/mL of hIL-1β) or vehicle (PBS alone) for 3 h.
6. After briefly rinsing the cells with PBS, lyse the cells and determine their luciferase activities with a *Renilla* luciferase assay kit (Promega) and a luminometer (Minilumat LB9506, Berthold).^8^
7. Determine the protein amounts with a Bradford reagent (Pierce) and normalize the optical intensities by the luminometer in form of a fold ratio, RLU (+)/RLU (−), where RLU (+) and RLU (−) are the luminescence intensities per a 1 μg cell lysate with and without a cytokine stimulation, respectively.

## Footnotes

1 Several web services are available for determining a hydrophilicity scale of a sequence including ExPASy Proteomics service (Swiss Institute of Bioinfomatics; SIB).

2 Precedent dissection sites in literatures may be referred upon choice of an optimal dissection site of a luciferase. The reason to choose a hydrophilic site is as follow: (i) many empirical studies reveal that a hydrophilic region can tolerate an insertion of the exogenous protein; (ii) hydrophilicity is required for the active site, because it is advantageous for recruiting substrates in aqua-phase; (iii) exposure of a hydrophobic region by dissection causes the decomposition in living cells [9].

3 The amino acid sequences in the interface region of the intein DnaE dominate the splicing affinity. If the splicing affinity is too strong, the background intensity is lifted by a spontaneous splicing reaction. The authors recommend to mutate the amino acids in the interface region to appropriately modulate the affinities if necessary. As shown in our previous study [6], modification of the interface sequences is helpful for weakening the excessive self-splicing reaction between the N- and C-terminal inteins.

4 Alternatively, the nuclear trafficking of cP50 in cells may be determined with a sucrose density gradient fractionation. The nucleus fraction may be separated by crushing the plasma membranes of the cells and centrifuging the homogenate at 600*g* from the other cell components.

5 A long incubation time causes overexpression of the indicator components, which raises the background intensities and worsens the corresponding signal-to-background ratios.

6 Murine TNF-α (mTNF-α) has a cross-reactivity with human TNF-α (hTNF-α).

7 Living mammalian cells consume 3 h to complete the splicing reaction and reach to a plateau.

8 The cell lysis time is important to obtain a better signal-to-noise ratio. The authors recommend researchers to optimize the lysis time according to their own assay kits. An excess lysis time causes a nonspecific protein splicing reaction between the split-luciferase fragments located in the cytosol and the nucleus by lysis of the nuclear membrane. It results in the background rise. The authors recommend less than 15 min of lysis time and a short centrifuge of the lysates (less than 1 min) in a moderate speed (3000 *g*) to eliminate non-lysed cells that cause the background rise.
